# Aqueous Humor Analyses in Patients with Diabetic Retinopathy Who Had Undergone Panretinal Photocoagulation

**DOI:** 10.1155/2022/1897344

**Published:** 2022-06-20

**Authors:** Jin-woo Kwon, Jusang Oh

**Affiliations:** Department of Ophthalmology, St. Vincent's Hospital, College of Medicine, The Catholic University of Korea, Seoul, Republic of Korea

## Abstract

**Purpose:**

To determine the associations between aqueous humor cytokine levels and the severity of diabetic retinopathy and the prior panretinal photocoagulation (PRP) status of patients with diabetic macular edema (DME).

**Methods:**

We divided 98 DME patients into those with nonproliferative diabetic retinopathy (NPDR), proliferative diabetic retinopathy (PDR), and PRP patients. We compared the concentrations of interleukin- (IL-) 1*β*, IL-6, IL-8, IL-10, and IL-17; placental growth factor (PlGF); and vascular endothelial growth factor (VEGF) in the aqueous humors. We subclassified PRP patients by the interval between PRP and aqueous sampling and analyzed the associations between aqueous cytokine levels and this interval.

**Results:**

The aqueous humor levels of IL-6, IL-8, VEGF, and PlGF were significantly higher in the PDR group than in the NPDR group. The PlGF and VEGF levels in the PDR group were significantly higher than those in the PRP group. On PRP subgroup analyses, patients who had undergone PRP within 6 months prior exhibited higher levels of VEGF, PlGF, and TNF-*α* than did those who had undergone PRP more than 12 months prior. The TNF-*α* level of the PRP subgroup treated within 6 months prior was significantly higher than that of the PDR group. Regression analyses showed that the levels of VEGF, PlGF, and TNF-*α* decreased significantly as the interval between PRP and aqueous sampling became longer.

**Conclusions:**

PDR patients exhibited higher concentrations of VEGF and certain inflammatory cytokines than did NPDR and PRP patients. In the latter patients, the intraocular VEGF and inflammatory cytokine levels fell gradually over time.

## 1. Introduction

Diabetic macular edema (DME) is one of the most significant causes of visual disturbance in patients with diabetic retinopathy (DR) [1, 2]. One meta-analysis reported that the overall prevalence of DR was 34.6%, that of proliferative DR (PDR) 6.96%, that of DME 6.81%, and that of vision-threatening DR 10.2% in patients with diabetes [3].

Of the various causes of DME, breakdown of the blood-retina barrier is characterized by a loss of pericytes and disruption of endothelial tight junctions induced by metabolic changes and inflammation [4, 5]. Many cytokines and cell types affect the neurovascular unit [4].

When the crucial role of vascular endothelial growth factor (VEGF) in this context was discovered and the effectiveness of anti-VEGF therapy in DR patients established, such therapy became the first-line treatment option for DME [6, 7]. Intravitreal steroids have also been widely used for several decades [8, 9].

Most studies of DME patients selected treatment options depending on the responsiveness to anti-VEGF preparations; steroids were prescribed for those with VEGF-refractory or chronic DME [10]. Optical coherence tomography (OCT) usefully predicts responsiveness and prognosis [11]. The use of aqueous humor biomarkers remains controversial; however, the levels thereof may reflect the pathogenesis and condition of DME patients [12, 13]. Some studies were of small sample size and enrolled very heterogeneous patients [14, 15]. More work is required; aqueous humor biomarkers might predict prognosis or allow customized treatment of selected DME patients. Therefore, we enrolled a relatively large number of patients when exploring changes in aqueous humor cytokine levels, and we determined whether such levels were affected by prior laser treatment.

## 2. Methods

The study protocol adhered to all relevant tenets of the Declaration of Helsinki and was approved by the institutional review/ethics board of the Catholic University of Korea. All participants gave written informed consent for use of their clinical records.

We enrolled treatment-naïve center-involving DME (ciDME) eyes of central subfield thickness (CST) ≥ 300 *μ*m from 2017 to 2020. Study participants were at least 18 years of age, had type II diabetes, and had received no anti-VEGF treatment or steroid treatments previously. The exclusion criteria included macular edema attributable to other causes. We also excluded eyes with any history of uveitis or intraocular surgery including cataract surgery and/or any laser treatments except panretinal photocoagulation (PRP). In patients who received PRP, we included eyes showing regression of NVE or NVD and no recurrent vitreous hemorrhage after the treatment. The PRP treatment used 20 ms pulse Pascal (Topcon Medical Laser Systems, Santa Clara, CA, USA) pattern-scanning laser photocoagulation with 3 × 3 multispot arrays and one burn width apart. Laser power was titrated (ranged from 200 to 500 mW) to apply a mild white burn with 200 *μ*m spot size using SuperQuad 160 lens according to ETDRS guidelines [16]. Burn distribution involved no closer than 2 disc diameters temporal to the fovea and 500 *μ*m nasal to disc and no further posterior than 1 burn within the temporal arcades. Numbers of final burns were ranged from 1800 to 2400.

We measured glycated hemoglobin levels and subjected all patients to ophthalmic examinations, including measurement of the best-corrected visual acuity (BCVA) and fundus examination. In addition, ultra-wide-field angiography was performed if necessary for DR classification. CST was measured using a Cirrus High-Definition OCT platform (Carl Zeiss Meditec, Dublin, CA, USA). Ellipsoid zone (EZ) disruptions were measured within 1,000 *μ*m in horizontal scans centered on the fovea. EZ disruption was graded as 0, no disruption; 1, focal disruption ≤ 200 *μ*m in length; and 2, disruption > 200 *μ*m in length [17].

### 2.1. Assay of Cytokines and Growth Factors

We measured the concentrations of IL-1*β*, IL-6, IL-8, IL-10, and IL-17; tumor necrosis factor- (TNF-) *α*; placental growth factor (PlGF); and VEGF in 75 *μ*L aliquots of aqueous humor. The detecting antibodies were immobilized on beads, and 75 *μ*L amounts of Calibrator Diluent RD6-52 (R&D Systems, Minneapolis, MN, USA) were added. The samples were incubated for 2 h after bead addition, for 1 h after antibody addition, and for 30 min after addition of the streptavidin-phycoerythrin reagent. Absorptions were read using the Luminex xMAP System (Luminex, Austin, TX, USA). All values under the lower limit of detection were assigned zero values.

### 2.2. Statistical Evaluation

Statistical analyses were performed with the aid of SPSS software for Windows ver. 21.0 (SPSS, Chicago, IL, USA). A one-way ANOVA, the Kruskal-Wallis test, the chi-squared test, and the Fisher exact test were used (as appropriate) to compare values or ratios. The post hoc Bonferroni correction was used when multiple statistical analyses were performed. The Spearman correlation test was employed when evaluating subgroup levels of cytokines by the interval between PRP and sampling.

## 3. Results

We enrolled 98 treatment-naïve ciDME eyes of 98 patients of mean age 56.79 ± 9.84 years (6 males and 42 females). In total, 43 patients had nonproliferative DR (NPDR, 43.88%), 17 had PDR (17.34%), and 38 had undergone PRP (38.78%). The mean BCVA (LogMAR) was 0.57 ± 0.30, and the mean CST was 418.40 ± 115.81 *μ*m at baseline. When classifying the DME morphology as cystoid macular edema (CME) or diffuse retinal thickening (DRT), 39 had CME and 59 DRT at baseline. The systemic and ocular characteristics of all patients are summarized in Table 1 by DR severity and PRP status.

**Figure 1:**
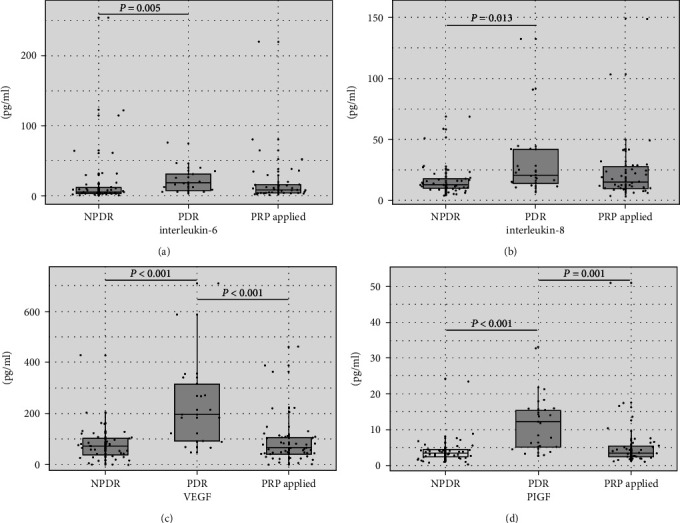
Box-and-jitter plots of the aqueous levels of IL-6 (a), IL-8 (b), VEGF (c), and PlGF (d) in NPDR, PDR, and post-PRP patients. The IL-6, IL-8, VEGF, and PlGF levels of PDR patients were significantly higher than those of NPDR patients. The VEGF and PlGF levels in the PDR group were higher than those in the post-PRP group. IL: interleukin; VEGF: vascular endothelial growth factor; PlGF: placental growth factor; NPDR: nonproliferative diabetic retinopathy; PDR: proliferative diabetic retinopathy; PRP: panretinal photocoagulation.

On post hoc analyses, the levels of IL-6, IL-8, VEGF, and PlGF in the PDR group were significantly higher than those in the NPDR group (*P* = 0.005, *P* = 0.013, *P* < 0.001, and *P* < 0.001, respectively), and the levels of VEGF and PlGF in the PDR group were higher than those in the PRP group (*P* < 0.001, *P* = 0.001) (Figure 1). We subdivided the PRP group by the interval between PRP and aqueous sampling; the systemic and ocular characteristics of the patients are summarized in Table 2. On post hoc analyses, the subgroup who had undergone PRP within 6 months prior to sampling exhibited significantly higher levels of VEGF, PlGF, and TNF-*α* than those who had undergone PRP more than 12 months prior (*P* < 0.001, *P* = 0.001, and *P* = 0.005, respectively).

**Figure 2:**
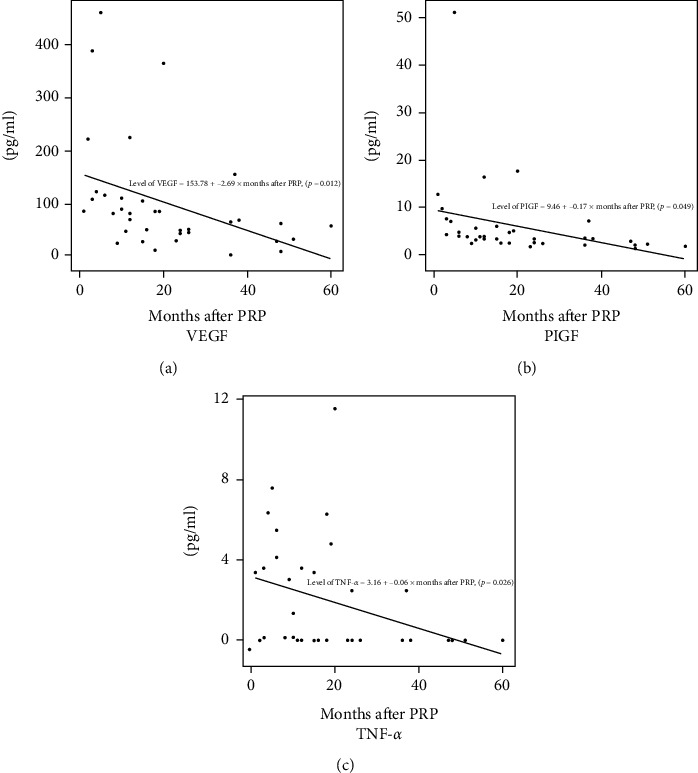
Univariate linear regression analyses by the aqueous levels of (a) VEGF, (b) PlGF, and (c) TNF-*α* by the interval between PRP and aqueous sampling. All levels were significantly negatively correlated with an increasing interval. VEGF: vascular endothelial growth factor; PlGF: placental growth factor; TNF: tumor necrosis factor; PRP: panretinal photocoagulation.

Univariate linear regression analyses were performed to explore the relationships between cytokine levels and the interval between PRP and sampling. The levels of VEGF, PlGF, and TNF-*α* decreased significantly when that interval was long rather than short (VEGF *r*_s_ = –2.69, *P* = 0.012; PlGF *r*_s_ = –0.17, *P* = 0.049; and TNF-*αr*_s_ = –0.06, *P* = 0.026, respectively; Figure 2). We compared the cytokine levels between the PRP subgroup who had been treated within 6 months prior and the PDR group. Only the TNF-*α* level differed significantly, being 3.82 ± 2.72 vs. 1.42 ± 2.22 pg/mL in the two sets of patients, respectively (*P* = 0.030).

## 4. Discussion

In this cross-sectional study, we found that the aqueous levels of certain proinflammatory interleukins and VEGF of PDR patients were significantly higher than those of NPDR patients and those who had undergone PRP. The TNF-*α* level early after PRP was significantly higher than that in the PDR group. PRP was associated with gradual decreases in the levels of aqueous VEGF and inflammatory cytokines over time.

The cytokine and VEGF levels well reflected the retinal and DR status. We assumed that PDR patients might exhibit higher levels of inflammatory cytokines and VEGF and that PRP would reduce these levels. Indeed, the PDR group evidenced the highest levels, and PRP gradually reduced these levels. Thus, aqueous humor measures may be valuable biomarkers of ocular status. However, aqueous humor parameters are affected not only by ocular status but also by intraocular injection or surgery; enrollment and grouping must be well controlled [18, 19]. We excluded patients with prior ocular surgery; we enrolled only treatment-naïve DME patients. We subgrouped PRP patients by the interval between PRP and aqueous sampling. We thus derived meaningful results.

PRP affects retinal and choroidal status [20, 21]. Few studies have explored how PRP influences the levels of intraocular cytokines or VEGF. A previous study reported that a PRP group exhibited a higher concentration of aqueous humor matrix metalloproteinase than controls [22] and another that PRP induced the synthesis of proinflammatory cytokines [23]. We found that, compared to the PDR group, the PRP group exhibited significantly lower levels of VEGF and PlGF. However, the more recent PRP group evidenced a significantly higher TNF-*α* level than the earlier PDR group. TNF-*α* is a potent proinflammatory cytokine that plays a key role in ocular inflammation [24]. Some studies have suggested that the aqueous TNF-*α* level is associated with uveitis and glaucoma [25, 26], but it is unclear whether this reflects DR severity [27]. An increased TNF-*α* level appeared to be associated with the extent of the inflammatory response after PRP, but further work is required.

Some reports found that PRP reduced the levels of intraocular VEGF and inflammatory cytokines and stabilized retinal status in DR patients [7, 28]. Other reports used fundus photography or OCT to evaluate serial retinal changes after PRP [29, 30]. However, no study has yet evaluated how long it might take to significantly reduce the levels of intraocular cytokines and VEGF. We found that patients who had undergone PRP more than 12 months prior to sampling exhibited PlGF, VEGF, and TNF-*α* levels that differed significantly from those in patients who had undergone PRP within 6 months prior to sampling. Linear regression analyses supported these findings. Thus, PRP affects intraocular status for a longer time than might be expected.

Our study has several limitations. Although we enrolled more patients than previous studies, the subgroup sizes were relatively small. Aqueous level of IL-6 in the subgroup that received PRP within 6 months prior to sampling was not the highest, unlike other inflammatory cytokines of VEGFs. Additionally, aqueous levels of other ILs were the highest in this subgroup but were not statistically significant. A larger sample size might have yielded different results. Also, this was a retrospective study; patients had been treated differently and the follow-up periods varied. In addition, various conditions in DME or PVD (posterior vitreous detachment) status could affect the aqueous profile of each patient [31]. We should have considered PVD status before planning this study using B-scans. We cannot make prognostic comments or analyze the responsiveness to DME treatments. In the future, we are planning to conduct a prospective, well-controlled study and animal study to compare aqueous profiles between before and after PRP treatment.

In conclusion, aqueous humor status well reflected retinal status. PDR patients exhibited higher concentrations of VEGF and certain inflammatory cytokines than did NPDR patients and those who had undergone PRP. The aqueous TNF-*α* level increased early after PRP. PRP was associated with gradual reductions in intraocular VEGF and inflammatory cytokine levels over time.

## Figures and Tables

**Table 1:** Demographic features depending on DMR staging.

		NPDR (*N* = 43)	PDR (*N* = 17)	PRP applied (*N* = 38)	*P* value
Systemic factors	Sex (male : female)	23 : 20	11 : 6	22 : 16	0.726
	Age (years)	59.53 ± 9.55	55.94 ± 10.45	56.63 ± 9.10	0.167
	Duration of diabetes	10.00 [3.50; 14.50]	7.00 [4.00; 10.00]	12.50 [6.00; 18.00]	0.008
	HbA1c (%)	7.80 [7.20; 8.30]	7.60 [6.70; 8.30]	7.30 [6.80; 7.80]	0.109
	Hypertension	22 (51.16%)	9 (52.94%)	22 (57.89%)	0.827
	Dyslipidemia	6 (13.95%)	3 (17.65%)	6 (15.79%)	0.934
	ESRD	1 (2.33%)	3 (17.65%)	4 (10.53%)	0.087
Concentrations of aqueous cytokines	IL-1 (pg/mL)	0.00 [0.00; 0.00]	0.00 [0.00; 0.00]	0.00 [0.00; 0.17]	0.338
	IL-2 (pg/mL)	0.00 [0.00; 0.00]	0.00 [0.00; 0.00]	0.00 [0.00; 3.62]	0.521
	IL-6 (pg/mL)	5.12 [3.66; 11.32]	18.40 [7.96; 31.31]	8.34 [4.29; 16.34]	0.016
	IL-8 (pg/mL)	12.79 [9.85; 17.84]	20.70 [14.10; 41.75]	14.85 [9.54; 28.43]	0.043
	IL-10 (pg/mL)	0.65 [0.26; 1.14]	0.53 [0.00; 1.34]	1.10 [0.50; 1.44]	0.233
	IL-17 (pg/mL)	0.54 [0.00; 2.16]	1.36 [0.54; 2.16]	1.36 [0.00; 2.16]	0.345
	TNF-*α* (pg/mL)	0.14 [0.00; 3.03]	0.00 [0.00; 2.32]	0.00 [0.00; 3.37]	0.852
	VEGF (pg/mL)	73.52 [38.19; 104.47]	196.12 [92.73; 316.82]	66.72 [43.02; 108.05]	<0.001
	PlGF (pg/mL)	3.55 [2.41; 4.40]	12.28 [5.17; 15.40]	3.41 [2.42; 5.58]	<0.001
OCT findings	Baseline CST	361.00 [326.50; 434.50]	382.00 [342.00; 432.00]	383.50 [355.00; 553.00]	0.088
	DME type (DRT : CME)	24 : 19	9 : 8	26 : 12	0.408
	EZD grade				
	0	24 (55.81%)	9 (52.94%)	16 (42.11%)	0.777
	1	9 (20.93%)	4 (23.53%)	12 (31.58%)	
	2	10 (23.26%)	4 (23.53%)	10 (26.32%)	

Values are expressed as the mean ± standard deviation or median and interquartile range, as appropriate. DMR: DM retinopathy; NPDR: nonproliferative diabetic retinopathy; PDR: proliferative diabetic retinopathy; PRP: panretinal photocoagulation; HbA1c: glycated hemoglobin; ESRD: end-stage renal disease; IL: interleukin; TNF: tumor necrosis factor; VEGF: vascular endothelial growth factor; PlGF: placental growth factor; OCT: optical coherence tomography; CST: central subfield thickness; DME: diabetic macular edema; DRT: diffuse retinal thickening; CME: cystoid macular edema; EZD: ellipsoid zone disruption.

**Table 2:** Characteristics of patients depending on interval between PRP and aqueous sampling.

		≤6 months after PRP (*N* = 8)	>6 months and ≤12 months after PRP (*N* = 8)	>12 months after PRP (*N* = 22)	*P* value
Systemic factors	Sex (male : female)	1 : 7	5 : 3	16 : 6	0.014
	Age (years)	54.00 ± 10.46	53.88 ± 7.70	58.59 ± 8.94	0.160
	Duration of diabetes	13.75 ± 6.67	7.25 ± 4.86	15.45 ± 8.99	0.309
	HbA1c (%)	7.00 [6.55; 7.55]	8.05 [6.95; 9.05]	7.30 [6.90; 7.70]	0.265
	Hypertension	5 (62.50%)	3 (37.50%)	14 (63.64%)	0.450
	Dyslipidemia	1 (12.50%)	0 (0.0%)	5 (22.73%)	0.581
	ESRD	2 (25.00%)	0 (0.0%)	2 (9.09%)	0.367
Concentrations of aqueous cytokines	IL-1 (pg/mL)	0.00 [0.00; 3.95]	0.00 [0.00; 0.21]	0.00 [0.00; 0.17]	0.661
	IL-2 (pg/mL)	4.60 [0.00; 12.27]	0.00 [0.00; 1.81]	0.00 [0.00; 0.00]	0.189
	IL-6 (pg/mL)	9.61 [4.92; 14.36]	21.01 [4.50; 50.03]	7.75 [3.70; 14.52]	0.665
	IL-8 (pg/mL)	19.13 [10.68; 26.87]	16.36 [10.48; 28.65]	13.30 [9.08; 23.49]	0.933
	IL-10 (pg/mL)	1.29 [0.90; 2.43]	0.94 [0.27; 1.55]	0.86 [0.26; 1.44]	0.459
	IL-17 (pg/mL)	2.16 [0.68; 5.75]	1.36 [0.00; 1.76]	0.54 [0.00; 2.16]	0.382
	TNF-*α* (pg/mL)	3.85 [1.76; 5.90]	0.14 [0.00; 2.19]	0.00 [0.00; 2.47]	0.011
	VEGF (pg/mL)	117.03 [109.59; 304.36]	79.36 [56.38; 97.55]	47.72 [27.05; 65.84]	0.001
	PlGF (pg/mL)	7.25 [4.44; 11.13]	3.74 [3.21; 4.66]	2.60 [2.19; 3.46]	0.002
OCT findings	Baseline CST	356.00 [339.00; 488.00]	415.00 [367.00; 594.00]	386.50 [359.00; 455.00]	0.293
	DME type (DRT:CME)	4 : 4	6 : 2	16 : 6	0.623
	EZD grade				
	0	4 (50.00%)	1 (12.50%)	11 (50.00%)	0.328
	1	3 (37.50%)	4 (50.00%)	5 (22.73%)	
	2	1 (12.50%)	3 (37.50%)	6 (27.27%)	

Values are expressed as the mean ± standard deviation or median and interquartile range, as appropriate. PRP: panretinal photocoagulation; HbA1c: glycated hemoglobin; ESRD: end-stage renal disease; IL: interleukin; TNF: tumor necrosis factor; VEGF: vascular endothelial growth factor; PlGF: placental growth factor; OCT: optical coherence tomography; CST: central subfield thickness; DME: diabetic macular edema; DRT: diffuse retinal thickening; CME: cystoid macular edema; EZD: ellipsoid zone disruption.

## Data Availability

The datasets generated during and/or analyzed during the current study are available from the corresponding author on reasonable request.
